# Optimal Sensor Placement for Leak Location in Water Distribution Networks Using Genetic Algorithms

**DOI:** 10.3390/s131114984

**Published:** 2013-11-04

**Authors:** Myrna V. Casillas, Vicenç Puig, Luis E. Garza-Castañón, Albert Rosich

**Affiliations:** 1 Supervision and Advanced Control Chair, Tecnológico de Monterrey, Campus Monterrey, Av. Eugenio Garza Sada 2501, Monterrey 64849, Mexico; E-Mails: mv.casillas.phd.mty@itesm.mx (M.V.C.); legarza@itesm.mx (L.E.G.-C.); 2 SAC Research Group, Institut de Robòtica i Informàtica Industrial (IRI-CSIC), Universitat Politècnica de Catalunya (UPC), Llorens i Artigues, 4-6, Barcelona 08028, Spain; 3 Interdisciplinary Centre for Security, Reliability and Trust, University of Luxembourg, 4, rue Alphonse Weicker L-2721, Luxembourg; E-Mail: albert.rosich@uni.lu

**Keywords:** water distribution networks, leak isolation, sensor placement, sensitivity analysis

## Abstract

This paper proposes a new sensor placement approach for leak location in water distribution networks (WDNs). The sensor placement problem is formulated as an integer optimization problem. The optimization criterion consists in minimizing the number of non-isolable leaks according to the isolability criteria introduced. Because of the large size and non-linear integer nature of the resulting optimization problem, genetic algorithms (GAs) are used as the solution approach. The obtained results are compared with a semi-exhaustive search method with higher computational effort, proving that GA allows one to find near-optimal solutions with less computational load. Moreover, three ways of increasing the robustness of the GA-based sensor placement method have been proposed using a time horizon analysis, a distance-based scoring and considering different leaks sizes. A great advantage of the proposed methodology is that it does not depend on the isolation method chosen by the user, as long as it is based on leak sensitivity analysis. Experiments in two networks allow us to evaluate the performance of the proposed approach.

## 1. Introduction

Leaks in water distribution networks are an issue of great concern for water utilities, strongly linked with operational costs and water resources savings. Continuous improvements in water loss management are being applied, and new technologies are developed to achieve higher levels of efficiency [1].

The traditional approach to leakage control is a passive one, whereby the leak is repaired only when it becomes visible. Recently, developed acoustic instruments [2] allow one to also locate invisible leaks, but unfortunately, their application over a large-scale water network is very expensive and time-consuming. A viable solution is to divide the network into a district metered area (DMA), where the flow and the pressure are measured, and to maintain a permanent leakage control system [3]. Then, leak detection in the DMA consists of monitoring flows at night, when customers demand is low and the leakage component is at its largest percentage over the flow. Therefore, practitioners monitor the DMA or groups of DMAs for detecting (and then repairing) leakages by analyzing the minimum night flow and, also, employ techniques to estimate the leakage level [1].

Regarding leak location methods for DMAs, several works have been proposed in the literature. For example, a review of transient-based leak detection methods is offered in [4] as a summary of current and past work. Model-based leak detection and isolation techniques have also been studied, starting with the seminal paper of Pudar and Liggett [5], which formulates the leak detection and isolation problem as a least-squares estimation problem. However, in such non-linear models, the parameter estimation of the water networks is not an easy task. Alternatively, in [6], a method based on pressure measurements and leak sensitivity analysis is proposed. This methodology consists in analyzing the residuals on-line, i.e., the difference between the measurements and their estimation using the network hydraulic models, regarding a given threshold that takes into account the model uncertainty and the noise. When some of the residuals violate their threshold, they are correlated against the leak sensitivity matrix in order to discover which possible leak is present. Although this approach presents satisfactory results under ideal conditions, its performance decreases in the presence of nodal demand uncertainty and noise in the measurements. An improved technique has recently been developed [7,8], where an extended time horizon analysis of pressure measurements is considered and a comparison between the performances depending on the metric used is performed.

Thus, the development of a sensor placement strategy has become an important research issue in recent years. Ideally, a sensor network should be configured to facilitate fault detection and maximize leak location performance. However, it is obvious that only a limited number of sensors can be installed inside a DMA, due to budget constraints. The main objectives of sensor placement are leak detectability, isolability and localization. Leak detectability is the ability of monitoring a variation in pressure due to a loss of water occurring in the network. Leak isolability concerns the capacity of distinguishing between two possible leak occurrences, whereas leak localization refers to finding the node where the leak is occurring. There are some works devoted to sensor placement for fault detection and isolation (FDI). Some approaches propose to locate sensors based on isolability criteria according to the study of structural matrices [9]. In [10], an optimization method based on binary integer linear programming searches for an optimal set of sensors for model-based FDI.

Each of the previously mentioned works is used in the general framework of FDI of dynamic systems. However, there are several contributions dedicated to sensor placement in water distribution networks. Most of the works have addressed the sensor placement problem regarding contamination monitoring. See, for example [11,12]; the problem of deploying sensors in a large water distribution network is considered in order to detect the malicious introduction of contaminants. On the other hand, less work has been done regarding sensor placement for leak location. In [13], a strategy based on isolability maximization allows one to optimally locate sensors for leak location based on the structural model of water network. Closer to our research, in [6], an optimal sensor placement for leak location is formulated as an integer programming problem. Recently, in [14], an entropy-based approach for the purposes of efficient and economically viable water loss incident detection was presented.

This paper proposes a new approach for sensor placement for leak location in DMAs that can be used with the projection-based location scheme proposed in [7,8]. The proposed approach is different from the algorithm presented in [6], since no binarization of the leak sensitivity matrix is used, being able to work directly using the numerical expression of this matrix. As shown in [7,8], leak isolation methods that use the leak sensitivity matrix without binarization lead to better performance. In particular, the method that computes the projection (angle) between the observed residuals and the columns of the leak sensitivity matrix is the one that performs the best. For this reason, the sensor placement approach developed in this paper uses the non-binarized leak sensitivity matrix and the projection-based leak isolation approach. The use of the non-binarized leak sensitivity matrix leads to completely reformulating the optimization problem solved by the algorithm presented in [6], since the isolability conditions will be completely different. In a binary context, two leaks are isolable if the corresponding columns of the binarized leak sensitivity matrix are different. However, in a non-binarized context, such criteria should be reformulated, as will be shown in this paper. As in [6], the sensor placement problem is finally formulated as an integer optimization problem. However, now, the optimization criterion consists in minimizing the number of non-isolable leaks according to the isolability criteria introduced. Because of the large size and non-linear integer nature of the resulting optimization problem, genetic algorithms (GAs) are used as the solution approach. The obtained results are compared with a semi-exhaustive search method with a higher computational cost, proving that GA allows one to find near-optimal solutions with less computational load. Another advantage is that the proposed sensor placement methodology does not depend on the isolation method chosen by the user, as long as it is based on leak sensitivity analysis. Moreover, since the numerical expression of the leak sensitivity matrix is used and this sensitivity depends on the leak size, among other factors, a robust sensor placement approach is also proposed. Experiments in two networks allow for evaluating the performance of the proposed approach.

The rest of the document is organized as follows: Section 2 presents the leak localization methodology in which our work is based. Section 3 describes the problem formulation. Sections 4 and 5 present the sensor placement algorithms proposed in this work, while in Section 6, we show the improvements performed to increase the robustness of the approach. Section 7 shows the application and the results obtained in a real water distribution network. Finally, Section 8 concludes this work.

## 2. Leak Location Methodology

The leak location methodology used in this paper has been introduced in [7,8], as an extension of the methodology proposed in [6]. This approach is summarized here, since it is the basis on top of which the sensor placement algorithm proposed in this paper will be formulated.

The leak location methodology aims to detect and isolate leaks in a DMA using pressure measurements and their estimation using the hydraulic network model. Let us consider a DMA with *m* demand nodes and *n* pressure sensors. The leak detection methodology is based on the computation of the residual vector **r** = [*r*_1_ … *r_n_*]*^T^*, where the residual, *r_i_* ∈ **r**, is the difference between the pressure measurements, *p_i_*, and its corresponding estimation, *p̂_i_*, obtained from the simulation of the hydraulic model with no leak, *i.e.*,:

(1) $$r_{i}=p_{i}-{\hat{p}}_{i}$$

for *i* = 1, … , *n*. Note that there is one residual for each pressure measurement available in DMA.

The leak isolation method relies on analyzing the residual vector (1) using sensitivity analysis, which is determined from the different effects on every pressure measurement caused by each possible leak at a time. To perform such sensitivity analysis, the following sensitivity vectors are derived from simulated leak scenarios [6]:

(2) $${\text{S}}_{j}=\left[\begin{array}{c} \frac{{\hat{p}}_{1}^{f_{j}}-{\hat{p}}_{1}}{f_{j}}\\ \vdots \\ \frac{{\hat{p}}_{n}^{f_{j}}-{\hat{p}}_{n}}{f_{j}} \end{array}\right]$$

for *j* = 1, ⋯ ,*m*, where ${\hat{p}}_{i}^{f_{j}}$ and *p̂_i_* are the pressure estimation obtained from the hydraulic DMA model simulation under the leak *f_j_* scenario and the leak-free scenario, respectively. More precisely, each simulated fault scenario is performed by injecting a leak of a magnitude of *f_j_* in the *j^t^^h^* DMA network node in order to compute the sensitivity vector (2). For the sake of simplicity and without loss of generality, *m* possible leaks (one for each node) have been assumed. Then, the leak isolation is based on the analysis of the residual vector, together with the sensitivity vectors, in order to determine which node has the highest risk of presenting a leakage. A variety of metrics can be used to perform this isolability analysis [15]. In this work, one of the methods presented in [7] based on projections between residual and sensitivity vectors is used. According to the mentioned study, this method presents the best performance for the location task. However, it is important to note that the sensor placement approach proposed in this paper could also be applied using any other leak location method based on sensitivity analysis (e.g., methods based on the other metrics in [7]).

Let **r** be the residual vector (1) obtained from the pressure sensors installed in the network. Its normalized projections, *ψ_j_*, onto each sensitivity vector are computed as:

(3) $$\psi_{j}=\frac{{\text{r}}^{T}{\text{s}}_{j}}{\left|\text{r}\right|\left|{\text{s}}_{j}\right|}$$

for *j* = 1, … , *m*. Then, the largest projection will determine the candidate node that presents a leak, i.e., a leak in node *k* is located if:

(4) $$\psi_{k}=\max(\psi_{1},\cdots ,\psi_{m})$$

## 3. Problem Formulation

The objective of this work is to develop an approach to place a given number of sensors, n, in a DMA of a water distribution networks (WDN) in order to obtain a sensor configuration with a maximized leak isolability performance for a given leak detection and isolated scheme. In this work, we use the method based on projections that has been presented in the previous section.

It should be noted that the length of the sensitivity and residual vectors that appear in Equations (1) and (2) corresponds to the number of sensors, *n*, installed in the network. In order to find a sensor configuration that presents maximum isolability performance regarding all the possible leak scenarios, the following residual vectors derived from simulated leak scenarios are computed:

(5) $${\text{r}}_{k}=\left[\begin{array}{c} {\hat{p}}_{1}^{f_{k}}-{\hat{p}}_{1}\\ \vdots \\ {\hat{p}}_{n}^{f_{k}}-{\hat{p}}_{n} \end{array}\right]$$

for *k* = 1, ⋯ ,*m*, where ${\hat{p}}_{i}^{f_{k}}$ and *p̂_i_* are the pressure estimation obtained from the hydraulic model simulation under the leak *f_k_* scenario and the leak-free scenario, respectively. Note that the magnitude of the leaks used to compute the sensitivity vectors in Equation (2) and the one used to compute the residual vectors in Equation (5) are chosen differently (*i.e.*, *f_j_* ≠ *f_k_*) in order to increase the robustness of the method. Taking into account the mentioned residual and sensitivity vectors, the sensitivity matrix, *S*, and the residual matrix, *R*, are constructed by concatenating all sensitivities and residuals as follows:

(6) $$S=\left[\begin{array}{ccc} {\text{s}}_{1} & \cdots & {\text{s}}_{m} \end{array}\right]$$

(7) $$R=\left[\begin{array}{ccc} {\text{r}}_{1} & \cdots & {\text{r}}_{m} \end{array}\right]$$

Note that the matrices, *S* and *R*, are computed assuming that all the nodes are measured.

To select a configuration with *n* sensors, the following binary vector is defined:

(8) $$\text{q}=\left[\begin{array}{ccc} q_{1} & \cdots & q_{m} \end{array}\right]$$

where *q_i_* = 1 if the pressure in the node, *i*, is measured, and *q_i_* = 0, otherwise (*i.e.*, the vector **q** denotes which sensors are installed). In turn, a diagonal matrix, *Q*(**q**), is constructed from the vector, **q**, as:

(9) $$Q(\text{q})=\text{diag}(q_{1},\cdots ,q_{m})$$

Then, the corresponding sensitivity and residual vectors can be determined as:

(10) $$\begin{array}{ll} {\text{s}}_{j}(\text{q})=Q(\text{q}){\text{s}}_{j}, & {\text{r}}_{k}(\text{q})=Q(\text{q}){\text{r}}_{k} \end{array}$$

for *j* = 1, … , *m*, where **s***_j_* and **r***_k_* are the sensitivity and residual vectors obtained with all nodes measured (*i.e.*, *m* = *n*, and both vectors, **s***_j_*, and the vectors, **r***_k_*, contain *m* elements each). Finally, the projections in Equation (3) can be computed depending on the sensors with respect to **q** as:

(11) $$\psi_{kj}(\text{q})=\frac{{\text{r}}_{k}^{T}Q(\text{q}){\text{s}}_{j}}{\left|Q(\text{q}){\text{r}}_{k}\right|\left|Q(\text{q}){\text{s}}_{j}\right|}$$

for *j* = 1,…, *m*. Note that the property *Q*(**q***^T^*)*Q*(**q**) = *Q*(**q**) has been used in Equation (11).

Now, we are able to compute the projection matrix, Ψ, as:

(12) $$\Psi (\text{q})=\left[\begin{array}{ccc} \psi_{11}(\text{q}) & \cdots & \psi_{1m}(\text{q})\\ \vdots & \ddots & \vdots \\ \psi_{m1}(\text{q}) & \cdots & \psi_{mm}(\text{q}) \end{array}\right]$$

In order to evaluate the quality of a sensor configuration regarding its capacity to locate a leak at node *i* ∈ {1, … , *m*}, and assuming the case of a single leak, the next error index is introduced:

(13) $$\varepsilon_{i}(\text{q})=\{\begin{array}{ll} 0 & \text{if}\;\psi_{ii}(\text{q})=\max(\psi_{i1}(\text{q}),\ldots ,\psi_{im}(\text{q}))\\ 1 & \text{otherwise}. \end{array}$$

This means that the error index *ε_i_* = 0, as long as the leak in node *i* is perfectly located, and *ε_i_* = 1, otherwise.

As the objective is to maximize the isolability regarding leaks in all network nodes, the error index that takes into account all the nodes leaks is computed as:

(14) $$\bar{\varepsilon }(\text{q})=\sum_{i=1}^{m}\frac{\varepsilon_{i}(\text{q})}{m}$$

We remark that *ε̄*(**q**) = 0, as long as a sensor configuration is chosen, such that all possible leaks can be perfectly located, and 100 · *ε̄*(**q**) is the percentage of incorrectly located leaks.

Based on the vector, **q**, and the extended error index, *ε̄*(**q**), the sensor placement problem is cast as an optimization problem formulated as:

(15) $$\begin{array}{c} \underset{\text{q}}{\min}\bar{\varepsilon }(\text{q})\\ s.t.\\ \overset{m}{\underset{i=1}{\text{∑}}}{\text{q}}_{i}=n \end{array}$$

where **q** is defined in Equation (8) and *n* ∈ {1,…, *m*} is the number of sensors we want to place.

***Remark***. It is important to note that the solution of the previous optimization algorithm provides the best sensor location when the size of the leak that we want to locate is close to the value used for evaluating residuals Equation (5). If the leak size is smaller or larger than this value, the optimal sensor location could vary. Moreover, the obtained leak isolation error could be larger than the minimum value obtained as the solution of the optimization problem Equation (15). In a later section, we will see how we propose to introduce some robustness (*i.e.*, against leak magnitude changes), improving the overall sensor placement method.

## 4. Semi-Exhaustive Search Approach

### 4.1. Semi-Exhaustive Search

As stated in Section 3, the problem of sensor placement involves finding an n-sensor configuration among a set of *m* candidate nodes. One trivial approach to solve the problem would be to check all the $\left(\begin{array}{c} m\\ n \end{array}\right)$ sensor configurations. However, this would result in a very high computational cost. Here, we propose the first algorithm as an alternative to this trivial methodology in order to ensure the optimal location in a benchmark network. This method involves the search for the best configuration based on every possible combination, but using lazy evaluation mechanisms to reduce the computation cost by discarding configurations as soon as we see that they cannot be candidates for the optimum.



**Algorithm 1** Sensor placement based on semi-exhaustive search.
**Require:** A sensitivity matrix, *S*, and a residual matrix, *R*. The number of sensors, *n*, the number of nodes, *m*, and a (*d* × *n*) matrix, *L*, where 
$d=\left(\begin{array}{c} m\\ n \end{array}\right)$, *i.e.*, each row is a possible combination of sensors position.**Ensure:** The optimal sensors configuration of index *k_min_* with error *ε̄_min_*.1:*min_NL_* ← *m*2:**for**
*k* = 1, … , *d*
**do**3: **q***^k^* ← *eval*_*Q*(*L^k^*) // *cf.*
Equation (8)4: *Ŝ^k^* ← *eval_S*(**q***^k^*, *S*); *R̂^k^* ← *eval_R*(**q***^k^*, *R*) // *cf.*
Equation (10)5: 
$nb_{NL}^{k}\leftarrow 0$6: **for**
*α* = 1, … ,*m*
**do**7:  
$\Psi_{\alpha \alpha }^{k}\leftarrow \text{eval}\_\Psi ({\hat{S}}^{k},{\hat{R}}^{k},\alpha )$ // *cf.*
Equations (11) and (12)8:  **for**
*β* = 1, … , *m*; *β* ≠ *α*
**do**9:   
$\Psi_{\alpha ,\beta }^{k}\leftarrow \text{eval}\_\Psi ({\hat{S}}^{k},{\hat{R}}^{k},\alpha ,\beta )$10:   **if**
$\Psi_{\alpha \beta }^{k}>\Psi_{\alpha \alpha }^{k}$
**then**11:    
$nb_{NL}^{k}\leftarrow nb_{NL}^{k}+1$12:    break13:   **end if**14:  **end for**15:  **if**
$nb_{NL}^{k}\geq min_{NL}$
**then**16:   break17:  **end if**18: **end for**19: **if**
$nb_{NL}^{k}<min_{NL}$
**then**20:   
$min_{NL}\leftarrow nb_{NL}^{k}$21:  *k_min_* = *k*22: **end if**23:**end for**24:
${\bar{\varepsilon }}_{min}=\frac{min_{NL}}{m}$


The method is described in Algorithm 1. The goal of this algorithm is to find the optimal sensor configuration, taking into account all the possible combinations of sensors and considering the method that will be used to perform the leak location. First, the algorithm initiates the minimum number of non-localizable (NL) leaks, *min_NL_*, found so far to *m* (line 1). Then, a loop is performed over each possible combination, *k*, of sensor configuration (line 2). The binary vector, q*^k^*, is evaluated, which allows one to compute the updated sensitivity and residual matrices, *i.e.*, *Ŝ^k^* and *R̂^k^*, respectively (lines 3 and 4), and the current number of NL leaks is initiated to be zero (line 5). Then, the algorithm checks for each potential leak, *α*, if it can be located with the current sensor configuration. It evaluates the element (*α*, *α*) of the matrix, Ψ, and for each other column, *β*, of row, *α*, it tests if the projection gives a higher score (line 10). If that is the case, then the number of NL leaks is augmented (line 11), and the other columns of the Ψ matrix do not need to be tested (line 12). When the number of NL leaks is higher than the minimum number of NL leaks found so far, *i.e.*, $nb_{NL}^{k}\geq \min_{NL}$, then the current configuration cannot be optimal, and the algorithm aborts the evaluation and continues with the next configuration (line 16), improving, in this way, the computational efficiency of the algorithm. Otherwise, the minimum number of NL leaks is updated by the current number of NL leaks (line 20) and the index of the configuration is taken as the best index found so far (line 21). This algorithm performs a semi-exhaustive search in the sense that all the configurations are considered, but useless computations are avoided as much as possible.

### 4.2. Hanoi Network Application Example

The semi-exhaustive approach was tested in the water network of Hanoi, Vietnam [16]. This benchmark has been used in several works [17,18], where the goal was to design or optimize the operation of a water network. The network consists of 31 demand nodes, one reservoir node and 34 pipes. The first test uses Algorithm 1 to compute the optimal location in the case of two sensors with *ε̄_min_* = *min_NL_*/*m*. The network model is simulated using EPANET [19]. To study the effect of the leak magnitude on the sensor placement algorithm, the leak magnitude is varied by changing the node emitter coefficient (*EC*) in EPANET from two to eight (*i.e.*, corresponding to leaks between 20 and 80 liters per second (lps)) and computing the resulting sensitivity and residual matrices, *S* and *R* (the *EC* of each node to be specified for individual leaks is given by *EC* = *w*/*p^p_exp_^* where *w* is the water flow, *p* is the fluid pressure and *p_exp_* is a fixed pressure exponent). Note that for this test network, these values are chosen in proportion to the demands of the network, in order to cause a perceptible effect in the pressure. Table 1 presents the leak isolation error index (see Equation (14)) obtained when *S* and *R* are computed using different *EC* values. It can be noticed that even in the worst case, the error index is lower than 0.2, meaning that less than 20% of the leaks (*i.e.*, six leaks) are not located in the right node. We can conclude that for a small network, the leak isolation errors, due to the unknown leak size, are small, even when installing just a few sensors. The diagonal elements of Table 1 are not computed, since they correspond to the ideal case where no uncertainties are considered and the minimum error is obviously zero.

As the second test, we perform the same experiment, but with three sensors. The results are shown in Table 2. First, we note that the error index, due to the unknown leak magnitude, is reduced when more sensors are installed. In both cases (with two and three sensors), the best configuration of sensors used to compute the error index is dependent on the combination of *ECs* used to compute the matrices, *S* and *R*. Thus, it is not possible to take a direct decision about the optimal sensor placement with these results. To mitigate this problem, we propose a post-treatment analysis to choose such placement. Since the network is small, when we change the sensitivities and the leak magnitudes, there are some configurations that are repeated many times. We will take advantage of such behavior and select the node configurations with the highest occurrence in the results of the semi-exhaustive search.

In order to choose an adequate combination of sensors, we count the occurrences of the configurations leading to the error indices in Tables 1 and 2 and look for those that are found the most. The idea is to find those configurations with the minimal error index that cover as many different leak magnitudes as possible. In the example of the Hanoi network, the three configurations with the highest occurrence in the case of the placement of two sensors are:

- Nodes {12, 21} with 16 occurrences
- Nodes {12,13} with 13 occurrences
- Nodes {7,12} with seven occurrences

and in the case of three sensors, the three configurations with highest occurrence are:

- Nodes {12,14, 21} with 22 occurrences
- Nodes {12, 21, 27} with 22 occurrences
- Nodes {12, 21, 29} with 18 occurrences

Table 3 gives the error indices averaged over each combination of residuals and sensitivities for the three best configurations, in the case of two and three sensors. Among these candidates, we consider the one that leads to the lowest error. It appears that in the case of two sensors, the optimal sensor configuration for the Hanoi network corresponds to the pair of nodes {12, 21}, whereas in the case of three sensors, the best configuration is obtained installing the sensors at the nodes {12,14, 21}.

## 5. Genetic Algorithms Approach

### 5.1. Introduction

Genetic algorithms (GAs) are well-known search and optimization tools based on the principles of natural genetics and natural selection [20,21]. Because of their broad applicability, ease of use and global perspective, GAs have been increasingly applied to various search and optimization problems in the recent past. Some fundamental ideas of genetics are borrowed and used artificially to construct search algorithms that are robust and require minimal problem information. GAs transform a population of individual objects, each with an associated fitness value, into a new generation of the population using the Darwinian principle of reproduction and survival of the fittest and analogs of naturally occurring genetic operations, such as crossover (sexual recombination) and mutation. Each individual in the population represents a possible solution to a given problem. The genetic algorithm attempts to find a very good (or the best) solution to the problem by genetically breeding the population of individuals over a series of generations.

The GAs can be used in the context of sensor placement in WDN in order to find near-optimal placement for leak location. In that case, a chromosome corresponds to the possible presence or absence of a sensor at a given node.

### 5.2. Algorithm Description

Here, the sensor placement problem formulated as an optimization problem in Section 3 is solved using genetic algorithms and implemented using the GA Toolbox of MATLAB. The GA needs to establish a function whose output involves an index to be minimized. In our case, this function corresponds to the evaluation of the error index computed in Equation (14) according to the computation of the projection matrix, as in Equation (12). This error depends on the number of maximum values in each row of the matrix that are not elements of the diagonal in the projection matrix.



**Algorithm 2** Sensor placement based on genetic algorithms
**Require:** A sensitivity matrix, *S*, and a residual matrix, *R*. The number of sensors, *n*, the number of nodes, *m*, and the maximum number of iterations, *d*.**Ensure:** A near-optimal sensors configuration, **q***_min_*, with error index *ε̄_min_*.1:*init* ← *InitVarGA*()2:*restrict* ← *SetRestrictions*
$\left(\sum_{i=1}^{m}{\text{q}}_{i}=n\right)$3:*z* ← *ChooseSeed*()4:**for**
*k* = 1, … , *d*
**do**5: Create *I^k^* matrix of size ((*z* + 1) × *m*), where each row is a random initialization, such that:
$\begin{array}{l} {\text{I}}_{\text{ij}|\text{i}\neq (\text{z}+1)}^{\text{k}}\leftarrow \{\begin{array}{cc} 1 & \text{if row}\;i\;\text{is with a sensor in node}\;j\\ 0 & \text{otherwise} \end{array}\\ {\text{I}}_{(\text{z}+1)\text{j}}^{\text{k}}\leftarrow \{\begin{array}{cc} \left\{\right\} & \text{if}\;k=1\\ {\text{q}}^{\text{k}-1} & \text{otherwise} \end{array} \end{array}$6: GA-based search:7: **Inputs:**
*init*, *restrict*, *R*, *S*, *I^k^*.8: **while** An optimization criterion is not reached **do**9:  q ← *getConfig*()10:  *Ŝ*(**q**) ← *eval_S*(**q**, *S*(**q**))  *R̂* (**q**) ← *eval_R*(**q**, *R*(**q**))11:  Ψ(**q**) ← *eval_*Ψ(*R̂*(**q**),*Ŝ*(**q**))12:  *ε*(**q**) ← *eval_ε*(Ψ(**q**)) // *cf.*
Equation (13)13:  
$\bar{\varepsilon }(\text{q})\leftarrow \underset{i}{\text{mean}}(\varepsilon_{i}(\text{q}))$ //*cf.*
Equation (14)14: **end while**15: Find {q*^k^*, *ε̄^k^*} such that 
${\bar{\varepsilon }}^{k}=\underset{\text{q}}{\min}(\bar{\varepsilon }(\text{q}))$16:**end for**17:Find {**q***_min_*, *ε̄_min_*} such that 
$\bar{\varepsilon }=\underset{k}{\min}({\bar{\varepsilon }}^{k})$


The pseudo-code of the algorithm is shown in Algorithm 2. First, we initialize the variables of the GA (line 1), including the number of generations, the bit string type population, the tolerance as 10^−10^ and the elite count as one, in order to save one of the previous results analyzed. Then, we declare the search restriction (line 2), being that the number of “ones” in the solution corresponds to the number of sensors to install, and a seed size, *z*, is chosen (line 3). This seed creates an initial matrix with random sensor positions, and every location delivered by the GA is tested according to the function declared in the algorithm. The sensor placement is based on the construction of binary vectors, where the presence of a “one” represents a sensor located in the correspondent node. This vector allows one to select the adequate rows of the matrices, *S* and *R*, in order to compute the projection matrix according to the selected nodes to be measured. Once we have this projection matrix, we look for the maximum value of each row of the matrix, expecting the highest to be in the diagonal position. If it occurs, it means that the leak index equal to the row in question can be located with the selected sensor configuration. Otherwise, the leak cannot be located using this configuration.

#### 5.2.1. Hanoi Network Application Example

The experiments performed in the Hanoi network presented in Section 4.2 and based on a semi-exhaustive search were computationally very demanding, despite the lazy evaluation mechanisms involved. For such a simple example, 465 configurations had to be tested for the case of two sensors with an average computation time of 3 s and 4, 495 configurations for the case of three sensors, which takes 15 s on average. This means that performing a semi-exhaustive search in a large network is not feasible, because of the computational complexity that would quickly lead to testing millions of possible combinations. This is the motivation for using GA. All the tests performed using semi-exhaustive search are reproduced using Algorithm 2 applied to the Hanoi network. The solutions found are the same as the ones obtained with the semi-exhaustive search, but with a computational time lower than 9 s per iteration and including three generations in each of them. All the experiments were performed in MATLAB, using a Windows 7 computer with a Pentium Dual Core processor of 2 GHz, memory (RAM) of 4 GB and a 64-bit operating system.

## 6. Robust Sensor Placement

### 6.1. Improving the Sensor Placement Robustness

In our experiments, despite Algorithm 2 providing efficiently optimal solutions (since they are consistent with the semi-exhaustive search), we have seen that the algorithm requires a post-treatment analysis in order to make an adequate sensor placement decision when uncertainty (e.g., about the unknown leak magnitude) is considered. Moreover, we know that this placement represents a near-optimal solution that works only for the time instant evaluated. In the following, we present three improvements that avoid any post-treatment and increase the robustness of the GA-based sensor placement.

#### 6.1.1. Time Horizon Analysis

In the semi-exhaustive search and GA-based algorithm that we have presented, we took into account a single instant of time for the analysis. However, the configuration that gives the minimum error index in the leak isolation can vary when the demand changes along a given period of time. To address this problem, it is possible to improve the tasks of leak detection and isolation by considering a time horizon, as suggested in [7]. Thus, in the following, we incorporate a time horizon in the evaluation function of the GA, with the objective of increasing the quality of the sensor placement for a better leak isolation within the network. Note that there is no restriction on the time horizon magnitude.

The GA evaluation function is modified in order to work with the mean projection along the time horizon instead of using a single instant of time. With such modification, the candidate leak node is obtained by looking at the maximum of the mean projection, Ψ̄(**q**), defined by:

(16) $$\bar{\Psi }(q)=\frac{1}{T}\sum_{t=1}^{t=T}\Psi^{t}(\text{q})$$

where Ψ*^t^*(**q**) is computed using Equation (12) and *T* represents the number of time samples associated with the time horizon.

#### 6.1.2. Distance-Based Scoring

In the optimization problem Equation (15), the error index function Equation (13) was set to zero each time a leak was located in the correct node and to one, otherwise. This binary scoring process treats all the leak nodes that are incorrectly located in the same way. However, it may be interesting to provide a more informative scoring that would consider if the location returned is close to the real leak position or not.

We propose to rely on topological distances (*i.e.*, the number of nodes in the shortest path from one node to another in the network) with respect to the real leak position for evaluate the scores. When the topological distance increases, we linearly increase the error scoring until a cut-off distance, *d_max_*, for which the node score is then set to one. Thus, Equation (13) is replaced by the scoring function:

(17) $$\varepsilon_{i}(\text{q})=\{\begin{array}{cc} \frac{d_{i}(\text{q})}{d_{\max}} & \text{if}\;d_{i}(\text{q})<d_{\max}\\ 1 & \text{otherwise} \end{array}$$

where *d_i_* represents the topological distance between the leak node, *i*, and the node, *j*, that corresponds to the biggest projection value, *i.e.*, *ψ_ij_*(**q**) = max(*ψ_i_*_1_ (**q**),…,*ψ_im_*(**q**)).

The cut-off distance, *d_max_*, can be chosen by the user. In our case, we propose a distance that depends on the network size. Let us consider a network with the shape of a 2D squared-grid made of *m* = (2*i* + 1)^2^ nodes. Then, the distance from the center node to the network border would be given by $\frac{1}{2}\sqrt{m}$. Following such a relationship between topological distance and number of nodes, we propose to set the cut-off distance, such that:

(18) $$d_{\max}=\frac{1}{2}\sqrt{m}$$

#### 6.1.3. Robustness Regarding Leak Magnitude Variations

The choice of sensor placement is affected by the leak magnitude taken to build the sensitivities. However, in real scenarios, this magnitude cannot be determined in advance. To improve the robustness of the results according to such parameter changes, we propose to incorporate sets of sensitivities and residuals in the evaluation function that are computed from different leak magnitudes. Assume that there are *L* leak magnitudes *l* ∈ {1**,** … ,*L*}, each one associated with a residual matrix, *R^l^*, and a sensitivity matrix, *S^l^*. Then, the number of possible couples, {*R^l^*^1^,*S^l^*^2^}, with *l*_1_,*l*_2_ ∈ {1,… ,L} would be *L*^2^. Among them, we discard couples {*R^l^*,*S^l^*} built from the same leak magnitude, since in realistic scenarios, the leak magnitude used to build the sensitivity matrix will not match the real leak magnitude from which is built the residual matrix. Furthermore, we discard couples {*R^l^*^1^,*S^l^*^2^} with *l*_1_ > *l*_2_, since Ψ(*R^l^*^1^,*S^l^*^2^) = Ψ(*R^l^*^1^,*S^l^*^2^)*^T^*, and thus, they would lead to the same error index. Therefore, we obtain a total of $\left(\begin{array}{c} L\\ 2 \end{array}\right)$ couples of residual and sensitivity matrices {*R*, *S*}. Finally, the error index that we computed in Equation (14) will be now evaluated, taking into account the average of the error indices computed for each of these couples:

(19) $$\bar{\varepsilon }(\text{q})=\frac{1}{m\left(\begin{array}{c} L\\ 2 \end{array}\right)}\overset{m}{\underset{i=1}{\text{∑}}}\overset{\left(\begin{array}{c} L\\ 2 \end{array}\right)}{\underset{c=1}{\text{∑}}}\varepsilon_{i}^{c}(\text{q})$$

### 6.2. Robust Sensor Placement Algorithm

The three steps presented above increase the robustness of the sensor placement method with respect to the experimental variations. These new procedures shown in Algorithm 3 modify the iteration steps performed by GA that were previously presented in Algorithm 2.

The method basically consists of *d* iterations of the GA. At each step, it starts with an initialization phase of the GA similar to the one performed in Algorithm 2 (line 1). Then, iterations within the GA search are performed until one of the GA stop criteria is reached (line 5). To build the evaluation function, a loop is executed over each possible combination of input residual and sensitivity matrices, according to the different leak magnitudes (line 7). For each combination, c, and for each time instant, *t* (line 8), the updated residual and sensitivity matrices, as well as the projection matrices are evaluated (line 9 to 11). Then, we compute Ψ*^c^*(**q**), *i.e.*, the mean of the projection matrix over each time instant (line 13) and the resulting error vector, *ϵ^c^*(**q**) (line 14). Next, the averaged error index is evaluated (line 16), and the GA searches for the configuration, **q***^k^*, with minimum error index *ε̄^k^* (line 18). Finally, we look among the *d* configurations returned by the GA for the one with the best score (line 20).

### 6.3. Application to the Hanoi Network

Algorithm 3 was applied to the Hanoi network. In our tests, we take *L* = *7* leak magnitudes (20, 30, 40, 50, 60, 70 and 80 liters per second), which results in 21 couples of residual and sensitivity matrices. Furthermore, we use a 24-h time horizon with pressure measurements every hour, which gives a total of *T* = 25 time steps. The algorithm performs five main iterations with two generations in each of them, while the seed size is made of 30 vectors in the initial population.

Since the network has 31 nodes, Equation (18) returns a cut-off distance of three nodes to compute the scoring error in Equation (17). Thus, leak nodes with topological distances of zero, one and two nodes have an error of zero, $\frac{1}{3}$ and $\frac{2}{3}$, respectively. Nodes with a higher distance have an error of one.

**Figure 1.**
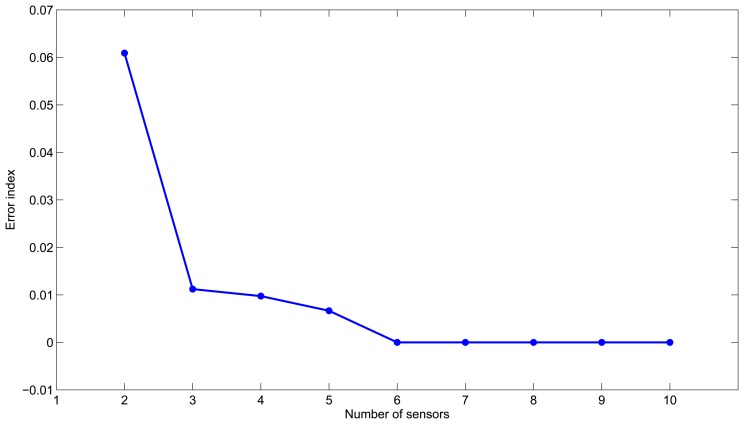
Minimum error index according to the number of sensors in the Hanoi network.

Algorithm 3 is executed varying the number of sensors from two to ten. Figure 1 shows the scoring error according to the number of sensors. We can see that after three sensors, the reduction of the error strongly decreases, and the use of additional sensors is not necessarily justified. This suggests that three sensors would be a good choice to have reliable leak detection and location. Furthermore, the best placement corresponds to nodes {12, 21} with an error index of 0.061 in the case of two sensors, and it corresponds to nodes {12,14, 21} with an error of 0.011 in the case of three sensors. Note that since this is a small example, these results give the same configurations as the ones found with both the semi-exhaustive search and Algorithm 2.



**Algorithm 3** Robust sensor placement based on genetic algorithms.
**Require:** A set of {*R^ct^*, *S^ct^*} couples of sensitivity and residual matrices with 
$c\in \{1,\ldots ,\left(\begin{array}{c} L\\ 2 \end{array}\right)\}$, representing the leak magnitudes, and *t* ∈ {1,…, *T*}, representing the samples of the time horizon. The number of sensors, *n*, the number of nodes, *m*, and the maximum number of iterations, *d*.**Ensure:** A robust near-optimal sensors configuration, **q***_min_*, with error index *ε̄_min_*.1:Compute *init*, *restrict*, and *z* // Steps 1 to 3 of Algorithm 2.2:**for**
*k* = 1, … , *d*
**do**3: Create *I^k^* matrix // Step 5 of Algorithm 2.4: GA-based search:5: **while** An optimization criterion is not reached **do**6:  **q** ← *getConfig*()7:  **for**
$c=1,\ldots ,\left(\begin{array}{c} L\\ 2 \end{array}\right)$
**do**8:   **for**
*t* = 1,… , *T*
**do**9:    *Ŝ^ct^*(**q**) ← *eval_S*(**q**,* S^ct^)*10:    *R̂^ct^*(**q**) ← *eval_R*(**q**, *R^ct^*)11:    Ψ*^ct^*(**q**) ← eval_Ψ(*R̂*
^ct^(**q**), *Ŝ^ct^* (**q**))12:   **end for**13:   
${\bar{\Psi }}^{c}(\text{q})\leftarrow \underset{t}{\text{mean}}(\Psi^{ct}(\text{q}))$ //*cf.*
Equation (16)14:   *ε^c^* (**q**) ← *eval_ε*(Ψ̄*^c^* (**q**)) //*cf.*
Equation (17)15:  **end for**16:  
$\bar{\varepsilon }(\text{q})\leftarrow \underset{i,c}{\text{mean}}(\varepsilon_{i}^{c}(\text{q}))$ //*cf.*
Equation (19).17: **end while**18: Find {**q***^k^*, *ε̄^k^*} such that 
${\bar{\varepsilon }}^{k}=\underset{\text{q}}{\min}(\bar{\varepsilon }(\text{q}))$19:**end for**20:Find {q*_min_*, *ε̄_min_*} such that 
$\bar{\varepsilon }=\underset{k}{\min}({\bar{\varepsilon }}^{k})$


## 7. Case Study: Limassol Network

**Figure 2.**
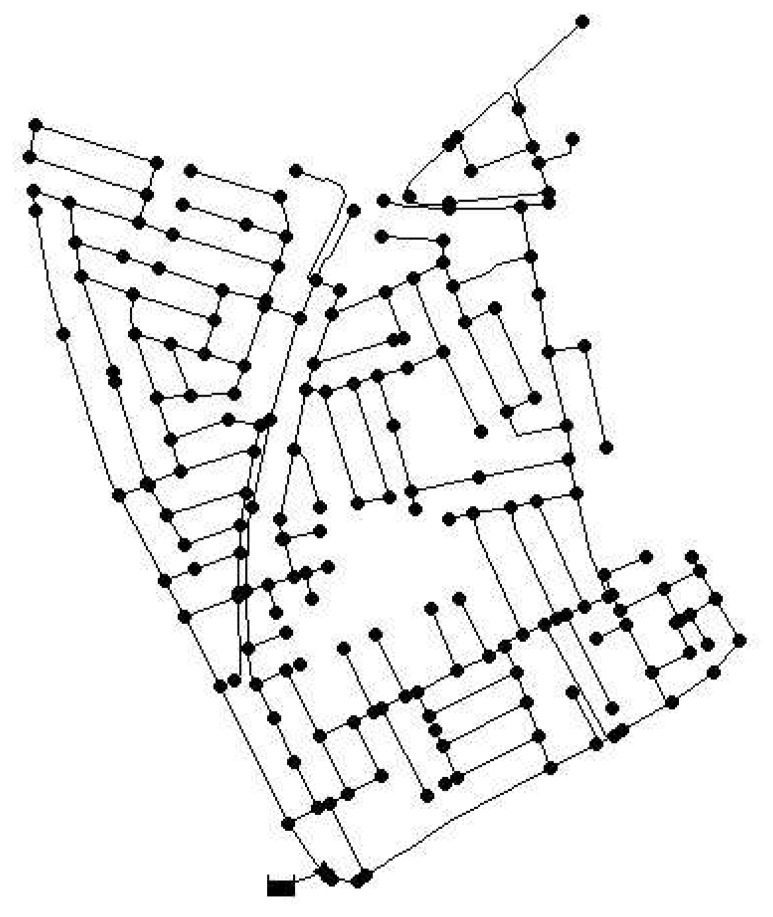
Water network in Limassol, Cyprus.

The methodologies presented in Algorithms 2 and 3 are applied to a real network. We used the Limassol network in Cyprus that has a total of 197 demand nodes and is represented in Figure 2. The network model is available in EPANET, as was the case for the Hanoi network. First, the semi-exhaustive algorithm is used to obtain three sensor placements that will serve as a reference to evaluate the performance of the GA approach. This algorithm is time-demanding in this case, since there are more than 1.2 × 10^6^ possible combinations of nodes to be considered. The computation time consumed by the semi-exhaustive search was approximately 60 h for the combination of sensitivity and residual chosen. This means that testing all the possible combinations of sensitivities and residuals is not feasible. The sensor placement problem is set up with *EC* = 0.25 (a leak of approximately 1.67 lps) for the sensitivities and *EC* = 0.20 (leak of approximately 1.3 lps) for the residuals. The best configuration obtained leads to placing sensors in nodes {82, 133, 157}, which gives an error index *ε̄_min_* = 0.258.

### 7.1. Application of the Sensor Placement Based on Genetic Algorithms

We apply Algorithm 2 for different types of residual and sensitivity matrices that are computed by varying the leak magnitudes within a given range. Here, the robustness improvements presented in Section 6.1 are not applied (they will be applied in the next subsection). The parameters for the genetic algorithm were selected after several trial and error tests. The initialization matrix was set with a size of 50 rows, and five iterations were allowed in order to increase the efficiency of the method with a maximum of five generations in each of them. The computation time was about 60 min. Compared to the time spent in the semi-exhaustive search, we can conclude that GA significantly reduces the time required to find a solution. Table 4 shows the nodes for each combination of EC value, whereas Table 5 shows the corresponding minimum error indices. From these tables, we can see that the algorithm finds different configurations depending on the leak magnitude (through EC) selected for the sensitivities and for the residuals. Such behavior was also occurring with the Hanoi network (*cf.* 4.2).

Thus, we perform a post-treatment analysis to decide what is the best sensor configuration for the network. This is based on the following tests:

- *Variation in the tested leak magnitude*: We compute the projection matrices for all the configurations found, taking into account the various combination of sensitivity and residual matrices corresponding to leak magnitudes changes.
- *Consideration of the sensors precision*: To take into account the limitation of the sensor precision, we truncate the two last decimals of the pressure measurements to compute the residual matrices.
- *Application of random noise in the measurements*: We include Gaussian white noise in the measurements with a mean amplitude corresponding to approximately 0.5% of the expected measurement, considering the technology of the pressure sensors used by the water company managing the network.

In order to select the adequate configuration of sensors, we propose performing the experiments described above and look for the combination with the smallest average error index along all the possible leak magnitudes and sensitivities to test. This criterion is analytically established by taking the minimum of the average error indices:

(20) $$\min(\frac{1}{L^{2}}\overset{L}{\underset{j=1}{\text{∑}}}\overset{L}{\underset{i=1}{\text{∑}}}\varepsilon^{ij})$$

where *L* is the number of leak magnitudes used and *ε^ij^* is the error index (*cf.* Equations (13) and (14)) obtained with the residual and sensitivity of the respective indices, *i* and *j*. In this way, the search for the best sensor placement is built as a *min-max* optimization problem.

Table 6 shows the errors induced by the three tests previously mentioned, as well as the total average error. From this table, we choose as the best sensor placement configuration the one with the lowest total average error.

Such a method gives the sensor placement at nodes {76, 133, 152}, which provides the lowest average error among the sets of sensor placements computed from the different combinations of residual and sensitivities. However, there is no guarantee that other sets would not lead to better results. Furthermore, this configuration was found based on a single time instant; thus, it is not robust to changes in the demand that would occur when considering a period of time. For these reasons and to get a more reliable solution, we prefer the method that includes the robustness improvements that we proposed in Section 6.1.

### 7.2. Application of the Robust Sensor Placement Method

We applied Algorithm 3 to the same Limassol, Cyprus network. We took *L* = 5 different leak magnitudes (one, 1.33, 1.66, two and 2.33 lps) that lead to 10 couples of sensitivity and residual matrices. The network data were analyzed taking into account a *T* = 24 h time horizon with pressure measurements every hour. Furthermore, the sensor noise (approximately 0.5% of the expected measurement as indicated above) was added to the computation of the residuals in order to increase the robustness of the method.

Since the network has 197 nodes, Equation (18) returns a cut-off distance of seven nodes to compute the scoring error in Equation (17). The algorithm performs five main iterations with two generations in each of them, while the seed size is made of 50 vectors in the initial population.

**Figure 3.**
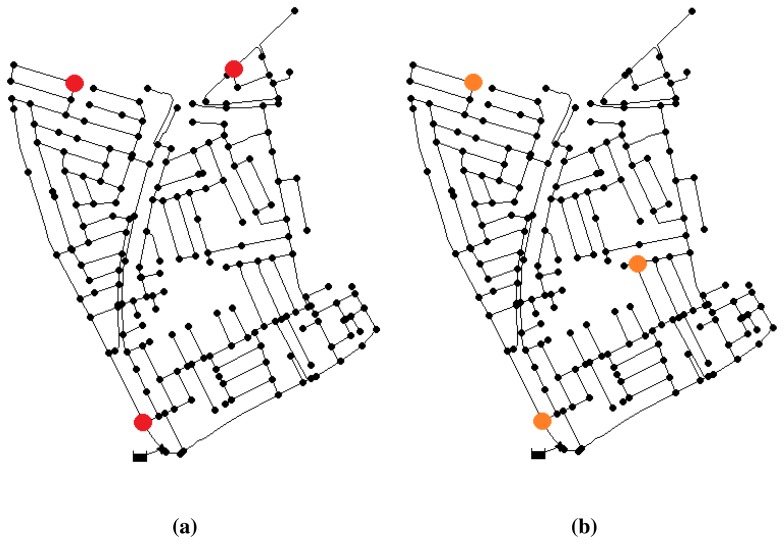
Near-optimal placement of three sensors in the Limassol network. (**a**) Sensor placement with noise of 0.5%; (**b**) sensor placement with noise of 2%.

After the iterations, the best result obtained corresponds to a sensor configuration of nodes {2, 75, 158}, with an error index of 0.302, which means an average distance of two nodes between the located node and that with the real leak. This placement is shown in Figure 3a.

A second test increasing the noise level up to 2% gives the best configuration at nodes {2, 75, 100} (Figure 3b) with an error index of 0.712. This means that leaks are located with an average of five nodes of topological distance to the real leak. Note that two of the nodes are repeated regarding the previous location. Consequently, despite the increased level of noise, the placement of the sensors has not been severally affected. Thus, it reassures the fact that the near-optimal solution will have a satisfactory behavior, even in different conditions.

It is important to note that these results comes from the integration within the GA of all the improvements of sensor placement robustness that we described in Section 6.1. Thus, contrary to what was done in the previous section when using Algorithm 2, there is no need to perform any post-treatment analysis to extract a robust solution. Integrating a time horizon, a more informative distance-based scoring and the possible variations of leak magnitude, our method provides a solution configuration for sensor placement with a higher level of confidence.

### 7.3. Practical Considerations

When moving the proposed sensor placement approach to the real network, the following practical considerations should be taken into account considering the previous experience in [6]:

- the DMA EPANET model should be recalibrated with real data obtained from the available sensors already installed in the network (typically at the flow entrance points) in order to minimize the errors due to model mismatch between the real and simulated network.
- the configuration of the internal valves should also be verified in order to assure that their positions in the real and simulated network are the same.
- the nodal demand should be estimated as well as possible using information from water consumption and tele-measurement devices, if available.
- leak size range that is considered, as well as sensor noise and precision should be characterized so that the robust sensor placement approach presented in Section 6 could be used in such a way that the installed sensors guarantee the minimal isolation error that is possible.

## 8. Conclusions

In this paper, we proposed a new approach to sensor placement for water distribution networks that maximizes leak isolability. The sensor placement problem has been formulated as a non-linear integer optimization problem. The optimization criterion is based on minimizing the number of non-isolable leaks according to the isolability criteria introduced. This approach is combined with a projection-based leak location scheme, but it could be easily adapted to any other sensitivity-based isolation scheme.

The first semi-exhaustive search method has been proposed that searches for the best configuration and relies on lazy evaluation mechanisms to reduce the computation cost. However, the computational effort remains too demanding for most realistic scenarios. Thus, we proposed to solve the optimization problem with GAs, which are known to work well in large-sized problems of a non-linear integer nature. We have seen that such approach allows us to find near-optimal solutions in an efficient way. We also highlighted that leak magnitude changes were impacting the resulting best sensor placement found by the GA algorithm, requiring a post-treatment analysis to tackle such a problem. Finally, we proposed three improvements that avoid any post-treatment and increase the robustness of the GA-based sensor placement. Experiments on two types of networks were performed to compare the different methods proposed in this paper. They demonstrate the relevance of the robust GA-based approach.

For future work, we would like to combine our robust GA-based approach with other methods to perform the leak isolation task. Furthermore, other types of optimization methods that provide some guarantee regarding the solution optimality could be investigated in the future. Enhancing the robustness of the sensor placement algorithm against additional sources of uncertainty, as in the model parameters or in nodal demand, will also be considered in future research.

## Figures and Tables

**Table 1.** Minimum error indices in the Hanoi network after placing two sensors. EC, emitter coefficient.

		**EC Used in Residuals**							

		2	3	4	5	6	7	8
**EC Used in Sensitivities**	2		0.032	0.032	0.129	0.129	0.129	0.193
	3	0.032		0.032	0.096	0.129	0.129	0.161
	4	0.064	0.032		0	0.064	0.096	0.129
	5	0.161	0.064	0.032		0.032	0.064	0.096
	6	0.161	0.129	0.064	0		0.032	0.096
	7	0.193	0.161	0.129	0.064	0.032		0
	8	0.193	0.193	0.161	0.129	0.064	0	

**Table 2.** Minimum error indices in Hanoi network with three sensors.

		**EC Used in Residuals**							

		2	3	4	5	6	7	8
**EC Used in Sensitivities**	2		0	0	0.032	0	0.032	0.032
	3	0		0	0	0.032	0	0.032
	4	0	0		0	0	0.032	0.032
	5	0.032	0	0		0	0.032	0.032
	6	0	0	0	0		0	0.032
	7	0.032	0.032	0.032	0	0		0
	8	0.032	0.032	0.032	0	0	0	

**Table 3.** Best configurations and corresponding error indices for different leak magnitudes in the Hanoi network.

**Two Placed Sensors**	(*n* = 2) **Case**	**Three Placed Sensors**	(*n* = 3) **Case**
**Configuration**	*ε̄*(**q**)	**Configuration**	*ε̄*(**q**)
{12, 21}	0.131	{12, 14, 21}	0.025
{12, 13}	0.133	{12, 21, 27}	0.028
{7, 12}	0.157	{12, 21, 29}	0.035

**Table 4.** Sensor configurations in Limassol network with three sensors.

		**Residuals EC**					

		0.15	0.2	0.25	0.3	0.35
**Sensitivities EC**	0.15		{40, 77 172}	{25, 77, 133}	{76, 133, 185}	{76, 133, 152}
	0.2	{76, 133, 152}		{76, 86, 152}	{77, 124, 152}	{76, 110, 173}
	0.25	{85, 156, 196}	{8, 76, 150}		{75, 116, 157}	{72, 115, 150}
	0.3	{72, 118, 163}	{76, 133, 141}	{77, 111, 150}		{75, 23, 152}
	0.35	{76, 128, 140}	{75, 120, 150}	{77, 115, 137}	{29, 112, 152}	

**Table 5.** Minimum error indices in the Limassol network for the configurations of Table 4.

		**Residuals EC**					

		0.15	0.2	0.25	0.3	0.35
**Sensitivities EC**	0.15		0.324	0.294	0.299	0.314
	0.2	0.299		0.284	0.279	0.294
	0.25	0.279	0.274		0.243	0.243
	0.3	0.309	0.279	0.263		0.258
	0.35	0.324	0.279	0.263	0.258	

**Table 6.** Averaged error indices for configurations of Table 4.

**Test**					

	**Configuration**	**Magnitude Change**	**Sensor Precision**	**Noise Addition**	**Total Average**
1	{75, 116, 157}	0.336	0.412	0.576	0.442
2	{85, 156, 196}	0.362	0.455	0.597	0.471
3	{72, 115, 150}	0.345	0.429	0.583	0.452
4	{76, 110, 173}	0.340	0.409	0.556	0.435
5	{77, 124, 152}	0.348	0.444	0.581	0.457
6	{76, 133, 152}	0.318	0.403	0.558	0.426
7	{76, 86, 152}	0.335	0.421	0.572	0.443
8	{25, 77, 133}	0.336	0.420	0.569	0.441
9	{76, 133, 185}	0.334	0.420	0.564	0.439
10	{40, 77, 172}	0.368	0.448	0.613	0.477
11	{**76, 133, 152**}	**0.318**	**0.403**	**0.546**	**0.422**
12	{8, 76, 150}	0.356	0.462	0.613	0.477
13	{72, 118, 163}	0.373	0.443	0.576	0.464
14	{76, 133, 141}	0.341	0.416	0.570	0.442
15	{76, 128, 140}	0.355	0.425	0.573	0.451
16	{75, 120, 150}	0.328	0.431	0.582	0.447
17	{77, 111, 150}	0.342	0.422	0.566	0.443
18	{77, 115, 137}	0.330	0.417	0.561	0.436
19	{75, 123, 152}	0.339	0.421	0.575	0.445
20	{29, 112, 152}	0.394	0.455	0.590	0.480
